# Supplementary figures and images for: Dysfunction of Calcyphosine-Like gene impairs retinal angiogenesis through the MYC axis and is associated with familial exudative vitreoretinopathy
Source: eLife. 2024 Sep 12;13:RP96907. doi: 10.7554/eLife.96907 (PMC11392532; doi:10.7554/eLife.96907)

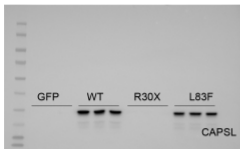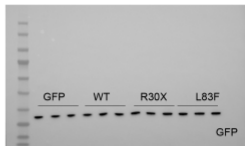

Supplement: Figure 1—source data 1. [file elife-96907-fig1-data1.zip › Figure 1-source data 1. Uncroped and labelled gels for Figure 1/Figure 1-source data1 uncroped and labeled gels for Figure 1.pdf]

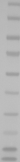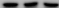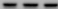

Supplement: Figure 1—source data 2. [file elife-96907-fig1-data2.zip › Figure 1-source data 2. Raw unedited gels for Figure 1/Figure 1-source data Raw unedited gel 1 for Figure 1.pdf]

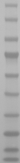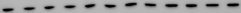

Supplement: Figure 1—source data 2. [file elife-96907-fig1-data2.zip › Figure 1-source data 2. Raw unedited gels for Figure 1/Figure 1-source data raw unedited gel2 for Figure 1.pdf]

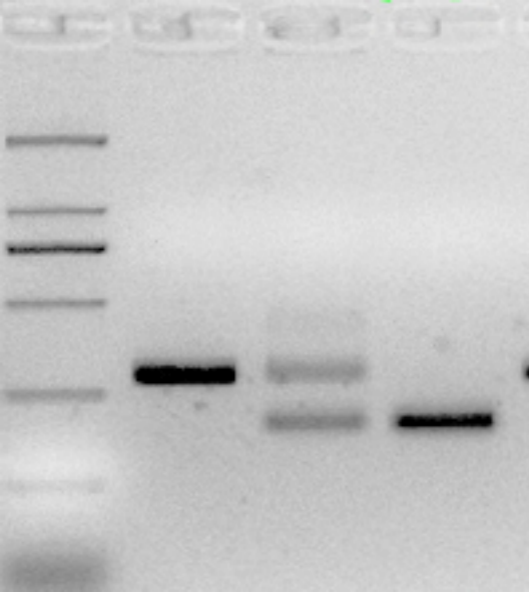

Supplement: Figure 2—figure supplement 1—source data 1. [file elife-96907-fig2-figsupp1-data1.zip › Figure 2-figure supplement 1-source data 1/Raw unedited gels 1.pdf]

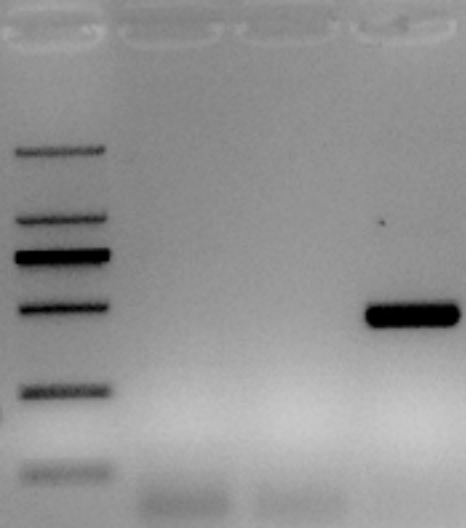

Supplement: Figure 2—figure supplement 1—source data 1. [file elife-96907-fig2-figsupp1-data1.zip › Figure 2-figure supplement 1-source data 1/Raw unedited gels 2.pdf]

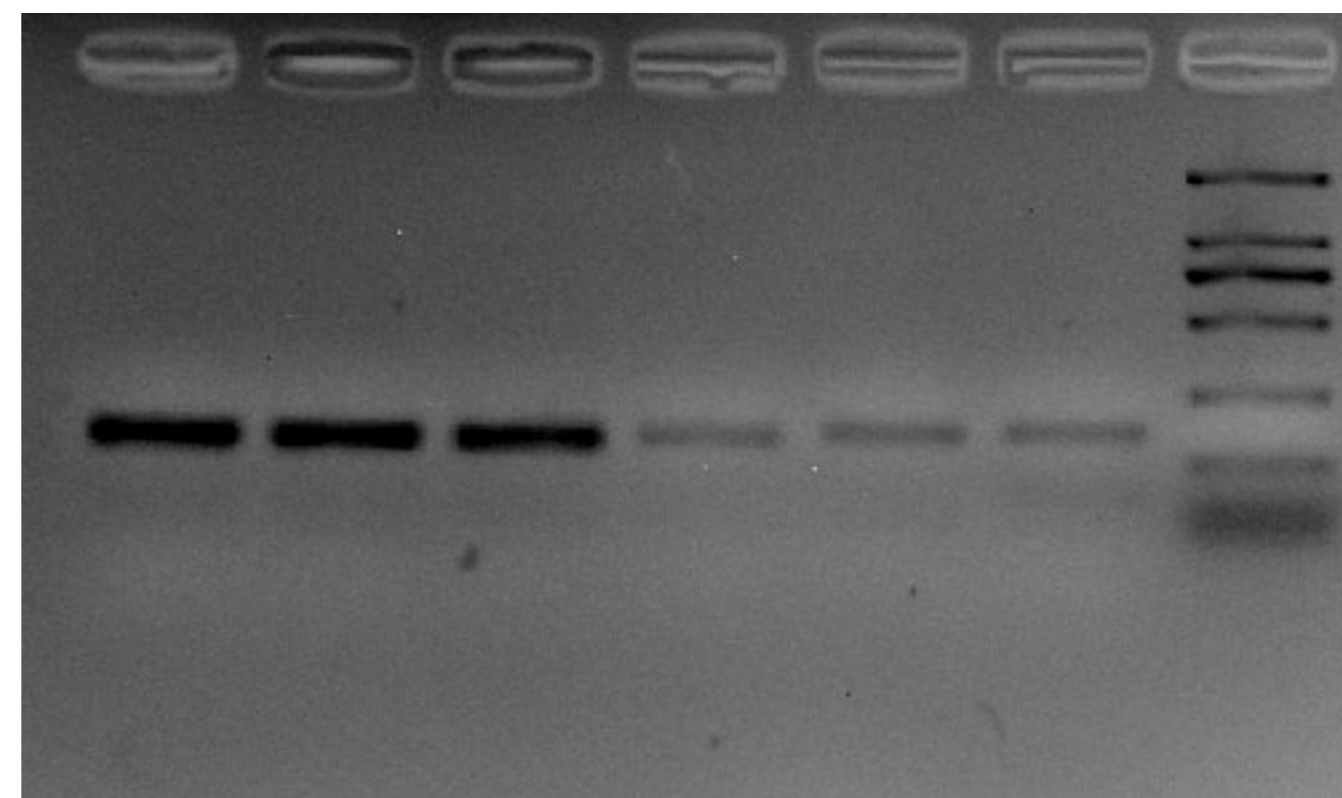

Supplement: Figure 2—figure supplement 1—source data 1. [file elife-96907-fig2-figsupp1-data1.zip › Figure 2-figure supplement 1-source data 1/Raw unedited gels 5.pdf]

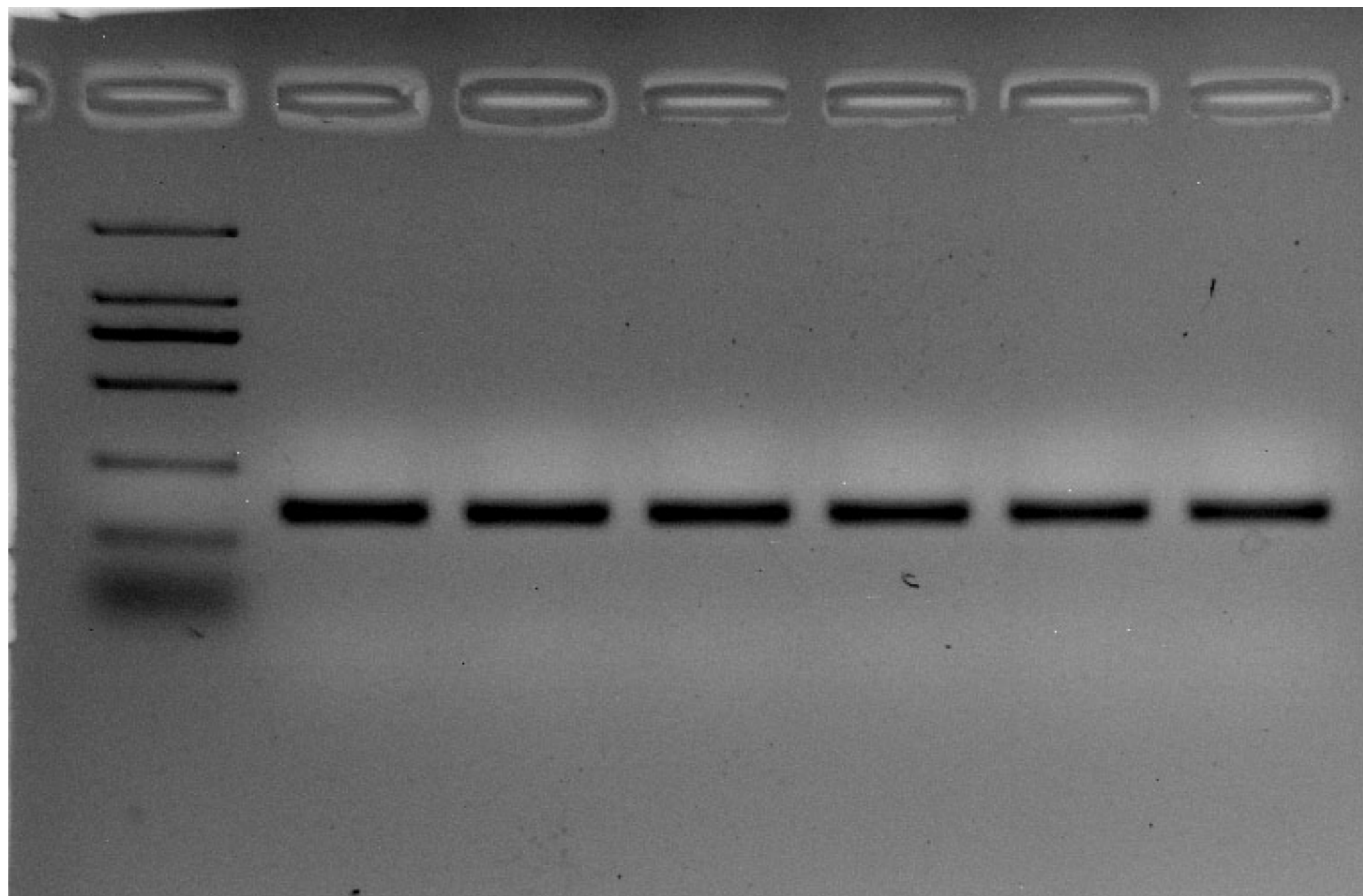

Supplement: Figure 2—figure supplement 1—source data 1. [file elife-96907-fig2-figsupp1-data1.zip › Figure 2-figure supplement 1-source data 1/Raw unedited gels 6.pdf]

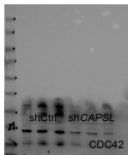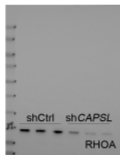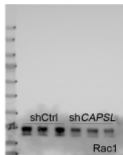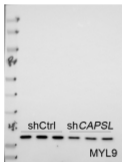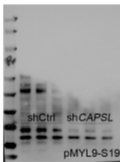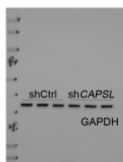

Supplement: Figure 5—source data 1. [file elife-96907-fig5-data1.zip › Figure 5-source data 1. Uncroped and labelled gels for Figure 5/Figure 5-source data1 uncroped and labeled gels for Figure 5.pdf]

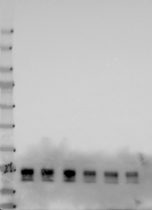

Supplement: Figure 5—source data 2. [file elife-96907-fig5-data2.zip › Figure 5-source data 2. Raw unedited gels for Figure 5/Figure 5-source data 3 Raw unedited gels for Figure 5.pdf]

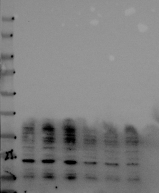

Supplement: Figure 5—source data 2. [file elife-96907-fig5-data2.zip › Figure 5-source data 2. Raw unedited gels for Figure 5/Figure 5-source data 1 Raw unedited gels for Figure 5.pdf]

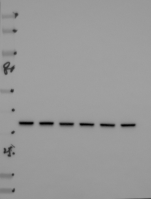

Supplement: Figure 5—source data 2. [file elife-96907-fig5-data2.zip › Figure 5-source data 2. Raw unedited gels for Figure 5/Figure 5-source data 6 Raw unedited gels for Figure 5.pdf]

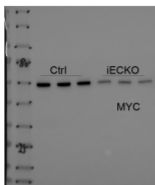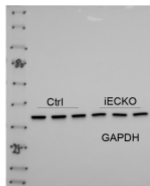

Supplement: Figure 6—figure supplement 2—source data 1. [file elife-96907-fig6-figsupp2-data1.zip › Figure 6-figure supplement 2-source data 1. Uncroped and labelled gels for Figure 6-figure supplement 2/uncroped and labeled gels.pdf]

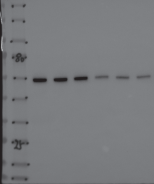

Supplement: Figure 6—figure supplement 2—source data 2. [file elife-96907-fig6-figsupp2-data2.zip › Figure 6-figure supplement 2-source data 2/Raw unedited gels 1.pdf]

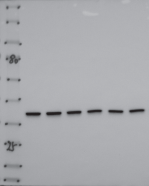

Supplement: Figure 6—figure supplement 2—source data 2. [file elife-96907-fig6-figsupp2-data2.zip › Figure 6-figure supplement 2-source data 2/Raw unedited gels 2.pdf]

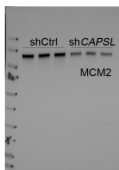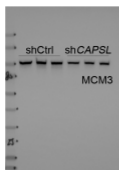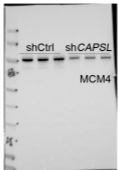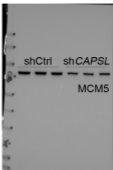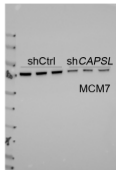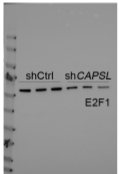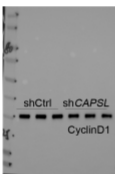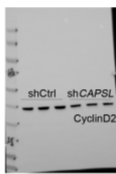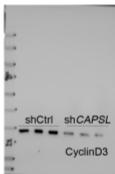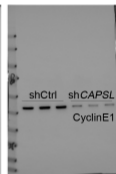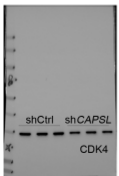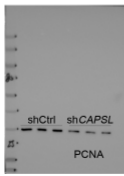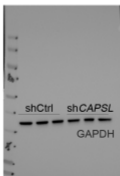

Supplement: Figure 7—source data 1. [file elife-96907-fig7-data1.zip › Figure 7-source data 1. Uncroped and labelled gels for Figure 7/Figure 7-source data2 uncroped and labeled gels for Figure 7.pdf]

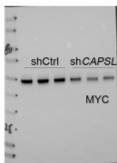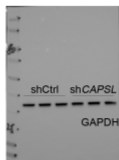

Supplement: Figure 7—source data 1. [file elife-96907-fig7-data1.zip › Figure 7-source data 1. Uncroped and labelled gels for Figure 7/Figure 7-source data1 uncroped and labeled gels for Figure 7.pdf]

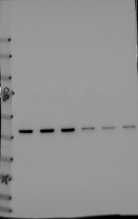

Supplement: Figure 7—source data 2. [file elife-96907-fig7-data2.zip › Figure 7-source data 2. Raw unedited gels for Figure 7/Figure 7-source data 12 Raw unedited gels for Figure 7.pdf]

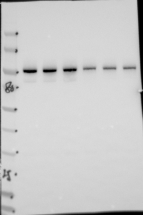

Supplement: Figure 7—source data 2. [file elife-96907-fig7-data2.zip › Figure 7-source data 2. Raw unedited gels for Figure 7/Figure 7-source data 5 Raw unedited gels for Figure 7.pdf]

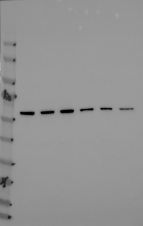

Supplement: Figure 7—source data 2. [file elife-96907-fig7-data2.zip › Figure 7-source data 2. Raw unedited gels for Figure 7/Figure 7-source data 8 Raw unedited gels for Figure 7.pdf]

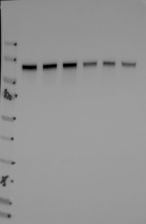

Supplement: Figure 7—source data 2. [file elife-96907-fig7-data2.zip › Figure 7-source data 2. Raw unedited gels for Figure 7/Figure 7-source data 3 Raw unedited gels for Figure 7.pdf]

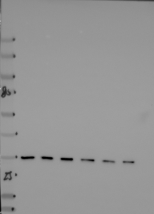

Supplement: Figure 7—source data 2. [file elife-96907-fig7-data2.zip › Figure 7-source data 2. Raw unedited gels for Figure 7/Figure 7-source data 14 Raw unedited gels for Figure 7.pdf]

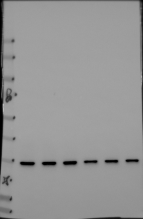

Supplement: Figure 7—source data 2. [file elife-96907-fig7-data2.zip › Figure 7-source data 2. Raw unedited gels for Figure 7/Figure 7-source data 13 Raw unedited gels for Figure 7.pdf]

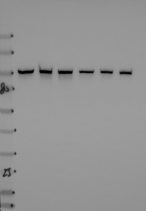

Supplement: Figure 7—source data 2. [file elife-96907-fig7-data2.zip › Figure 7-source data 2. Raw unedited gels for Figure 7/Figure 7-source data 4 Raw unedited gels for Figure 7.pdf]

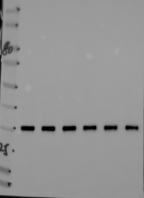

Supplement: Figure 7—source data 2. [file elife-96907-fig7-data2.zip › Figure 7-source data 2. Raw unedited gels for Figure 7/Figure 7-source data 9 Raw unedited gels for Figure 7.pdf]

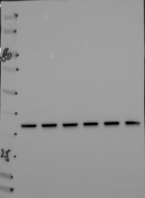

Supplement: Figure 7—source data 2. [file elife-96907-fig7-data2.zip › Figure 7-source data 2. Raw unedited gels for Figure 7/Figure 7-source data 2 Raw unedited gels for Figure 7.pdf]

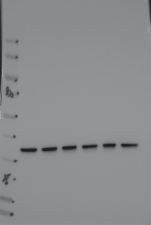

Supplement: Figure 7—source data 2. [file elife-96907-fig7-data2.zip › Figure 7-source data 2. Raw unedited gels for Figure 7/Figure 7-source data 15 Raw unedited gels for Figure 7.pdf]

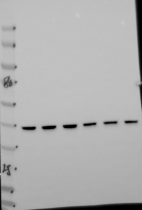

Supplement: Figure 7—source data 2. [file elife-96907-fig7-data2.zip › Figure 7-source data 2. Raw unedited gels for Figure 7/Figure 7-source data 10 Raw unedited gels for Figure 7.pdf]

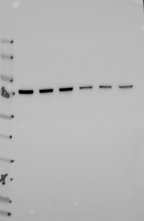

Supplement: Figure 7—source data 2. [file elife-96907-fig7-data2.zip › Figure 7-source data 2. Raw unedited gels for Figure 7/Figure 7-source data 7 Raw unedited gels for Figure 7.pdf]

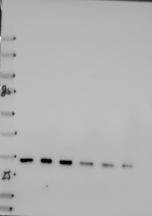

Supplement: Figure 7—source data 2. [file elife-96907-fig7-data2.zip › Figure 7-source data 2. Raw unedited gels for Figure 7/Figure 7-source data 11 Raw unedited gels for Figure 7.pdf]

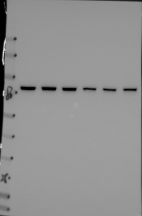

Supplement: Figure 7—source data 2. [file elife-96907-fig7-data2.zip › Figure 7-source data 2. Raw unedited gels for Figure 7/Figure 7-source data 6 Raw unedited gels for Figure 7.pdf]
