# Supplementary material for: Dysfunction of Calcyphosine-Like gene impairs retinal angiogenesis through the MYC axis and is associated with familial exudative vitreoretinopathy
Source: eLife. 2024 Sep 12;13:RP96907. doi: 10.7554/eLife.96907 (PMC11392532; doi:10.7554/eLife.96907)
Supplement: Figure 2—figure supplement 1—source data 1. [file elife-96907-fig2-figsupp1-data1.zip › Figure 2-figure supplement 1-source data 1/Raw unedited gels 4.pdf]

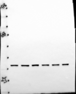
25
