# Supplementary material for: Dysfunction of Calcyphosine-Like gene impairs retinal angiogenesis through the MYC axis and is associated with familial exudative vitreoretinopathy
Source: eLife. 2024 Sep 12;13:RP96907. doi: 10.7554/eLife.96907 (PMC11392532; doi:10.7554/eLife.96907)
Supplement: Figure 7—source data 2. [file elife-96907-fig7-data2.zip › Figure 7-source data 2. Raw unedited gels for Figure 7/Figure 7-source data 1 Raw unedited gels for Figure 7.pdf]

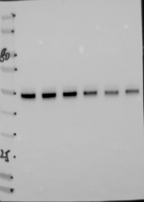
80
25
