# Supplementary material for: Dysfunction of Calcyphosine-Like gene impairs retinal angiogenesis through the MYC axis and is associated with familial exudative vitreoretinopathy
Source: eLife. 2024 Sep 12;13:RP96907. doi: 10.7554/eLife.96907 (PMC11392532; doi:10.7554/eLife.96907)

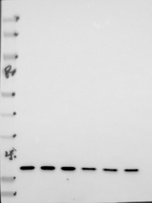

Supplement: Figure 5—source data 2. [file Figure 5-source data 4 Raw unedited gels for Figure 5.pdf › Figure 5-source data 2. Raw unedited gels for Figure 5/Figure 5-source data 4 Raw unedited gels for Figure 5.pdf]
